# The Disinfection of Books

**Published:** 1898-01

**Authors:** 


### THE DISINFECTION OF BOOKS.

At a recent discussion of this subject Dr. John S. Billings, the director of the New York Public Library, said, in part:

"At an investigation made by the State Board in Iowa three years ago it was found that six cases of scarlet fever were undoubtedly communicated through circulating library books. Other diseases may be communicated in the same way. There is not much danger of this from the edges of cards, although they are foul and filthy, and undoubtedly filled with bacteria, more especially with those of skin diseases. In order to prevent any possibility of the diseases through books it is necessary to destroy the bacteria.

"We cannot use the method of applying 210 degrees Fahrenheit of dry heat. That would curl the leaves and injure the bindings. Much less can we apply 212 degrees Fahrenheit of wet heat. We cannot apply a solution of corrosive sublimate or zinc chloride. The fumes of burning sulphur are both inadequate and undesirable.

"Two years ago, when in Philadelphia, I suggested to one of the fellows in the laboratory that it would be desirable to seek a new method. Formaline was known to be a destroyer of bacteria, and he set to work on that. This can be purchased in a drug-store, or may be made by burning methyl alcohol. A saucer of formaline, a book which had been infected with the bacteria of diphtheria, another with scarlet fever, and another with erysipelas were placed under a bell jar. The experiment showed that one cubic centimetre of formaline to 300 cubic centimetres of space would thoroughly disinfect any book in fifteen minutes."

